# Antibiotic Resistance and Virulence of Extraintestinal Pathogenic *Escherichia coli* (ExPEC) Vary According to Molecular Types

**DOI:** 10.3389/fmicb.2020.598305

**Published:** 2020-11-25

**Authors:** Yitao Duan, Huihui Gao, Liyang Zheng, Shuangqing Liu, Yang Cao, Siyuan Zhu, Zhenzhe Wu, Hongqiang Ren, Daqing Mao, Yi Luo

**Affiliations:** ^1^College of Environmental Science and Engineering, Ministry of Education Key Laboratory of Pollution Processes and Environmental Criteria, Nankai University, Tianjin, China; ^2^School of Medicine, Nankai University, Tianjin, China; ^3^Department of Clinical Laboratory, The Second Hospital of Tianjin Medical University, Tianjin, China; ^4^State Key Laboratory of Pollution Control and Resource Reuse, School of the Environment, Nanjing University, Nanjing, China

**Keywords:** extraintestinal pathogenic *Escherichia coli*, CH typing, multilocus sequence typing, resistance, virulence

## Abstract

Extraintestinal pathogenic *Escherichia coli* (ExPEC) can cause many human extraintestinal infections. Resistance and virulence of ExPEC are inextricably linked to its phylogenetic background. However, studies on type-specific distribution of resistance and virulence and the connection between resistance/virulence and molecular typing are lacking. Here, 411 ExPEC strains were collected and characterized using antimicrobial susceptibility testing and molecular typing. Among these, 74 representative strains were selected for whole genome sequencing and the *Galleria mellonella* killing assay. CH40-30-ST131, CH37-27-ST405, CH40-41-ST131, and CH13-5-ST12 isolates had high resistance rates to all antimicrobials tested. *Bla*_CTX–M_ played a significant role in the β-lactam resistance of ExPEC isolates. CH14-64-ST1193, CH40-30-ST131, and CH35-27-ST69 isolates were highly virulent in the *G. mellonella* model. Virulence factors (VFs) involved in adherence (*papB*, *papI*, *papX*, and *fimA*), autotransporter (*sat*), invasion (*aslA*, *kpsD*), iron uptake (except for *entD*), or toxin (*senB*) might be responsible for pathogenicity *in vivo*. Specific antibiotic resistance genes (ARGs) or VFs were prevalent in specific types of strains, including *papB*, *papI*, *fimA*, *sat*, *kpsD*, *senB*, and aerobactin genes in CH14-64-ST1193 isolates; *bla*_CTX–M–__15_, *aac(6′)-Ib-cr*, *papB*, *papI*, *sat*, *iucA*, *iucB*, *iucC*, *chuT*, *chuX*, and *shuU* in CH40-30-ST131 isolates; *tetB* in CH35-27-ST69 and CH13-5-ST12 isolates. Type distribution also differed by VF score. CH37-27-ST405 and CH26-5-ST38 isolates carried more ARGs and VFs indicating that they had a high resistance and virulence potential. This study demonstrates the type-specific distribution of resistance and virulence thus providing a basis for further research, prevention and treatment of ExPEC infections.

## Introduction

Extraintestinal pathogenic *Escherichia coli* (ExPEC) is one of the most common bacterial pathogens isolated from clinical specimens. It causes urinary tract infection, bacteremia, neonatal meningitis, and other infections at non-intestinal sites (Biran and Ron, 2018). Extensive use of antibiotics has led to a significant increase in antimicrobial resistance (especially against third-generation cephalosporins and fluoroquinolones) in ExPEC isolates severely affecting its effective treatment (WHO, 2019). ExPEC strains have a great impact on public health and economic burden due to the high percentage of ExPEC infections and its antimicrobial resistance.

Extraintestinal pathogenic *Escherichia coli* as an opportunistic pathogen, often causes infection in immunocompromised individuals, which relies primarily on antimicrobials as a first line of defense against infection. Virulence and antimicrobial resistance in ExPEC play an important role in the infection process. Resistance and virulence are inextricably linked to the phylogenetic background of the strain. Most ExPEC isolates belong to phylogenetic group B2 and to a lesser extent to group D, whereas most commensal isolates belong to groups A and B1 (Pitout, 2012). Isolates from group B2 demonstrated a high frequency of virulence factors (VFs) while group A strains displayed a low prevalence of VFs (Salipante et al., 2015). Up to 30% of all ExPEC, 60–90% of fluoroquinolone-resistant ExPEC, and 40–80% of ExPEC producing extended-spectrum β-lactamase (ESBL) belong to ST131 (Pitout and DeVinney, 2017). Within the ST131 isolates *bla*_CTX–M–__15_ and *aac(6′)-Ib-cr* were more prevalent in the H30-Rx sublineage (Peirano and Pitout, 2014). These 12 VFs (*iha*, *fimH*, *sat*, *astA*, *fyuA*, *iutA*, *kpsM II-K2*, *kpsM II-K5*, *usp*, *traT*, *ompT*, and *malX*) were significantly more prevalent in ST131 than in non-ST131 isolates (Johnson et al., 2010). With the exception of ST131 isolates, there are less studies on the characterization of antibiotic resistance and virulence of ExPEC belonging to other molecular types. Multilocus sequence typing (MLST) is the most common method of characterizing ExPEC-associated clonal lineages based on the sequencing of seven housekeeping genes (Manges et al., 2019). High-resolution CH (*fumC*/*fimH*) typing was also developed for identifying the population structure of ExPEC based on the sequencing of *fumC* and *fimH* (Weissman et al., 2012). And specific CH types correspond to specific STs with 95% accuracy (Roer et al., 2018). Although molecular typing, antibiotic resistance, and virulence of ExPEC have been detected and analyzed in some studies (Matsumura et al., 2017; Nüesch-Inderbinen et al., 2017; Bozcal et al., 2018; La Combe et al., 2019), the complete analysis of antibiotic resistance genes (ARGs) and VFs using genome sequencing is limited, and the comprehensive analysis and connection between antibiotic resistance/virulence and molecular typing are also limited or lacking. In this study, we conducted a systematic study of ExPEC to investigate the specific distribution of antibiotic resistance and virulence with regards to molecular types. We performed antimicrobial susceptibility testing, CH or MLST, screening for acquired ARGs and VFs using genome sequencing, and *Galleria mellonella* killing assay on ExPEC isolates. The study shows that antibiotic resistance and virulence of ExPEC strains differ according to molecular types. It provides a basis for further research targeting prevention and treatment.

## Materials and Methods

### Bacterial Isolates and Antimicrobial Susceptibility Testing

A total of 411 non-duplicate ExPEC isolates were obtained from clinical samples (Supplementary Table S5) of patients in the Second Hospital of Tianjin Medical University (Tianjin, China) in two successive periods (2011–2012 and 2018). Antimicrobial susceptibility testing and ESBL detection were evaluated using the Vitek 2 automated system (Sysmex-bioMérieux, Marcy I’Etoile, France). The tested antimicrobials, which are commonly used in clinical practice, included amikacin, gentamicin, amoxillin/clavulanic acid, ampicillin, ampicillin/sulbactam, piperacillin, piperacillin/tazobactam, aztreonam, cefatriaxone, cefazolin, cefepime, cefoperazone/sulbactam, cefotaxime, cefoxitin, ceftazidime, cefuroxime, ertapenem, imipenem, meropenem, trimethoprim/sulfamethoxazole, nitrofurantoin, ciprofloxacin, levofloxacin, minocycline, tetracycline, and tigecycline. *E. coli* ATCC 25922 was used as a control strain. Interpretation of the results was performed using the guidelines from the Clinical & Laboratory Standards Institute (CLSI, 2019). Intermediate and resistant results were analyzed as resistant. Multidrug-resistant (MDR) isolates were those resistant to ≥3 of the following drug classes: aminoglycosides, beta-lactams, trimethoprim/sulfamethoxazole, nitrofurantoin, fluoroquinolones, and tetracyclines. The resistance score was defined using the number of resistant antimicrobial agents for each isolate. A score of 1 indicates resistant; 0.5, intermediary; and 0, sensitive.

### Detection of Clonal Groups

Clonal groups were defined using CH (*fumC*/*fimH*) typing as described by Weissman et al. (2012). For *fimH*-null isolates (isolates without *fimH*), MLST was performed according to the protocol described by Wirth et al. (2006). Briefly, overnight cultured ExPEC isolates were heated to 100°C for 10 min for crude DNA extraction. The two (*fumC* and *fimH*) or seven (*adk*, *fumC*, *gyrB*, *icd*, *mdh*, *purA*, and *recA*) housekeeping genes were amplified by PCR. The amplified PCR products were then sequenced, and the results were analyzed using a Web tool for CH typing^1^ (CHTyper 1.0) or sequence type (ST) (MLST 2.0)^2^.

### Whole-Genome Sequencing and Sequence Analysis

Apart from 7, 2, and 19 isolates respectively in CH11-54, CH26-65, and “Others” group, about one-quarter of the isolates in each group were also sampled equidistantly between the lowest and highest resistance rates in each group (Supplementary Table S1). A total of 74 representative isolates were selected for whole-genome sequencing. They were subjected to DNA extraction and purification using a Bacteria genomic DNA kit (CWbiotech, Beijing, China) following the manufacturer’s instructions. A total amount of 1 μg DNA was used for the generation of sequencing libraries using NEBNext Ultra DNA Library Prep Kit for Illumina (NEB, United States) following manufacturer’s recommendations. The library was then sent to Novogene (Tianjin, China) for genome sequencing on an Illumina HiSeq Xten platform using a 150 bp paired-end strategy. Acquired ARGs carried by the isolates were analyzed using KmerResistance v 2.0 (Clausen et al., 2016) with raw reads. KMA v1.2.0 (Clausen et al., 2018) was used to detect VFs by aligning raw reads directly against the Virulence Factors of Pathogenic Bacteria Database (VFDB; “core dataset” downloaded on 21 June 2020) (Liu et al., 2019). Results were reported for ≥90% nucleotide identity, ≥90% coverage of the query, and a sequence depth of ≥10×. ARG and VF scores were defined as the number of unique ARG and VF detected for each isolate, respectively. STs of the strains were analyzed from raw reads using MLST v 2.0 (Larsen et al., 2012), with results reported for thresholds of 100% nucleotide identity, 100% coverage of the query, and a sequence depth of >50×. Raw reads were assembled into scaffolds using SPAdes v 3.13.1 (Nurk et al., 2013). The major ExPEC phylogenetic group (A, B1, B2, C, D, E, and F) was determined by analyzing the assembled scaffolds using the online service ClermonTyping^3^. The sequence data were deposited in NCBI Sequence Read Archive under the accession number SAMN14395432- SAMN14395505.

### Infection of *G. mellonella* Larvae

The 74 representative strains (Supplementary Table S1) were also used for the *G. mellonella* killing assay. Overnight cultures of ExPEC isolates were washed using phosphate-buffered saline (PBS, pH 7.0) and further adjusted with PBS to concentrations of 1 × 10^7^ colony-forming units (CFU)/mL. For each strain, 10 larvae were injected respectively with 10 μL of bacterial suspension (Alghoribi et al., 2014). In addition, there were three different control groups (10 larvae per group). The first control group was injected with *E. coli* DH5α to evaluate effects of non-pathogenic bacterial infection, the second group was injected with PBS to measure effects of physical injury, and the third group was not manipulated. Larvae were then incubated at 37°C in the dark and monitored for an additional 96 h. We recorded the number of larvae killed. All the experiments were replicated three times.

### Statistical Analysis

Comparisons between groups were analyzed using Kruskal–Wallis test or Mann–Whitney *U*-test for continuous variables and chi-square or Fisher’s exact test for categorical variables. A two-sided *P* value <0.05 was considered statistically significant. All statistical analyses were performed using IBM SPSS statistics version 25.0 (IBM Corp., Armonk, NY, United States).

## Results

### Molecular Typing and Sources of ExPEC

Molecular typing identified 91 CH types, 12 STs, and 11 unknown types in the 411 ExPEC isolates (Supplementary Table S1). The most prevalent molecular type was CH14-64, which was present in one-tenth of the isolates (43/411). Ten other prevalent molecular types were CH40-30, CH35-27, CH11-54, CH37-27, CH40-41, CH26-5, CH26-65, CH38-27, ST648 (*fimH*-null), and CH13-5, which accounted for 9.7, 5.6, 4.4, 4.1, 3.9, 3.2, 2.9, 2.9, 2.7, and 2.4% of the isolates, respectively. According to the phylogenetic groups and STs of 74 sequenced strains sampled from the 411 ExPEC isolates (Supplementary Table S1), we could infer some CH-ST correlations with phylogenetic groups of the different strains in the cohort, including CH14-64-ST1193, CH40-30-ST131, CH40-41-ST131, CH38-27-ST95, and CH13-5-ST12 belonging to group B2; CH35-27-ST69 and CH37-27-ST405, and CH26-5-ST38 belonging to group D; CH11-54 belonging to group F; and ST648 (*fimH*-null) belonging to group A.

Most of the 411 isolates were from urine (63.5%), sputum (15.6%), and blood (10%) samples (Supplementary Table S5). Compared with non-CH14-64 isolates, more CH14-64 isolates were from urine, which suggested that this type strain was a major contributor to urinary tract infection. However, fewer CH40-30 isolates were from urine compared with non-CH40-30 isolates. Interestingly, 9 out of 10 CH13-5 isolates were from sputum samples of infants with bronchopneumonia (2011.12-2012.04), which might be strongly implicated in the prevalence of pediatric bronchopneumonia during the winter and spring. Type distribution differed by sources of ExPEC.

### Antimicrobial Resistance

Extraintestinal pathogenic *Escherichia coli* isolates exhibited high resistance rates to ampicillin (91.1%), piperacillin (85.6%), cefazolin (82.7%), cefuroxime (67.6%), cefatriaxone (63.2%), cefotaxime (62.5%), tetracycline (80.5%), ciprofloxacin (71.7%), levofloxacin (67.1%), and trimethoprim/sulfamethoxazole (70.7%). However, piperacillin/tazobactam (14.5%), cefoperazone/sulbactam (16%), carbapenems [ertapenem (2.5%), imipenem (2.3%), and meropenem (2.3%)], tigecycline (0, data not shown), amikacin (6.2%), and nitrofurantoin (10.1%) exhibited good activity (>80% susceptible) against ExPEC isolates. Type distribution also differed by resistance phenotype, resistance score, MDR, and ESBL phenotype (Table 1). CH40-30-ST131, CH37-27-ST405, CH40-41-ST131, and CH13-5-ST12 isolates displayed high prevalence of resistance to third-generation cephalosporins [cefatriaxone (85–100%), cefotaxime, (80.6–100%), ceftazidime (50–65%), except for 20% CH13-5], MDR (75–100%), ESBL phenotype (63.6–100%), and high resistance score (10.3–13). CH14-64-ST1193, CH40-30-ST131, CH37-27-ST405, and ST648 (*fimH*-null) had high resistance rates to fluoroquinolones (ciprofloxacin or levofloxacin, 94.1–100%).

**TABLE 1:** Antimicrobial resistance according to CH or ST types among the 411 ExPEC isolates.

**Characteristic**	**No. (%) of isolates**													***P***
	**All (*n* = 411)**	**CH14-64 (*n* = 43)**	**CH40-30 (*n* = 40)**	**CH35-27 (*n* = 23)**	**CH11-54 (*n* = 18)**	**CH37-27 (*n* = 17)**	**CH40-41 (*n* = 16)**	**CH26-5 (*n* = 13)**	**CH26-65 (*n* = 12)**	**CH38-27 (*n* = 12)**	**ST648^a^ (*n* = 11)**	**CH13-5 (*n* = 10)**	**Others^b^ (*n* = 196)**	
Antimicrobial resistance^c^														
Amikacin (*n* = 356)^d^	22 (6.2)	3 (7)	3 (9.4)	0	0	2 (15.4)	1 (6.7)	0	2 (22.2)	1 (8.3)	1 (11.1)	0	9 (5.4)	0.370
Gentamicin (*n* = 297)^d^	168 (56.6)	15 (53.6)	25 (75.8)	8 (61.5)	5 (41.7)	11 (68.8)	10 (83.3)	3 (37.5)	7 (77.8)	8 (72.7)	5 (71.4)	0	71 (51.4)	0.001
Amoxillin/clavulanic acid (*n* = 285)^d^	180 (63.2)	15 (51.7)	22 (77.3)	10 (52.6)	6 (54.5)	12 (100)	10 (90.9)	5 (71.4)	5 (71.4)	8 (66.7)	4 (66.7)	8 (80)	75 (57.3)	0.040
Ampicillin (*n* = 292)^d^	266 (91.1)	31 (93.9)	29 (100)	14 (93.3)	8 (100)	12 (100)	14 (100)	9 (100)	9 (100)	9 (75)	7 (100)	10 (100)	114 (85.1)	0.124
Ampicillin/sulbactam (*n* = 219)^d^	141 (64.4)	15 (65.2)	16 (69.6)	5 (50)	2 (40)	12 (100)	10 (100)	3 (60)	3 (42.9)	5 (45.5)	3 (75)	2 (20)	65 (65.7)	0.001
Piperacillin (*n* = 215)^d^	184 (85.6)	18 (90)	23 (95.8)	8 (72.7)	4 (66.7)	12 (100)	11 (100)	6 (85.7)	5 (100)	7 (63.6)	5 (100)	9 (90)	76 (81.7)	0.125
Piperacillin/tazobactam (*n* = 393)^d^	57 (14.5)	2 (4.7)	9 (22.5)	3 (13.6)	6 (35.3)	2 (12.5)	1 (6.7)	1 (10)	2 (18.2)	0	0	0	31 (16.6)	0.084
Aztreonam (*n* = 295)^d^	130 (44.1)	12 (33.3)	19 (67.9)	4 (22.2)	2 (25)	11 (84.6)	9 (60)	1 (12.5)	5 (62.5)	6 (54.5)	4 (44.4)	4 (40)	53 (40.5)	0.001
Cefatriaxone (*n* = 400)^d^	253 (63.2)	18 (46.2)	34 (85)	8 (34.8)	12 (66.7)	16 (94.1)	12 (92.3)	9 (75)	7 (58.3)	6 (50)	5 (55.6)	10 (100)	116 (59.5)	<0.001
Cefazolin (*n* = 254)^d^	210 (82.7)	16 (66.7)	27 (93.1)	11 (78.6)	6 (75)	12 (100)	11 (100)	7 (100)	6 (100)	7 (58.3)	5 (100)	10 (100)	92 (79.3)	0.019
Cefepime (*n* = 411)^d^	209 (50.9)	16 (37.2)	26 (65)	5 (21.7)	9 (50)	14 (82.4)	11 (68.8)	6 (46.2)	7 (58.3)	5 (41.7)	7 (63.6)	7 (70)	96 (49)	0.006
Cefoperazone/sulbactam (*n* = 357)^d^	57 (16)	2 (4.8)	3 (8.8)	2 (9.5)	1 (9.1)	3 (23.1)	3 (20)	2 (20)	4 (36.4)	2 (16.7)	2 (22.2)	0	33 (19.5)	0.158
Cefotaxime (*n* = 317)^d^	198 (62.5)	15 (41.7)	25 (80.6)	5 (27.8)	6 (50)	12 (92.3)	12 (100)	8 (72.7)	4 (57.1)	6 (50)	4 (57.1)	10 (100)	91 (61.5)	<0.001
Cefoxitin (*n* = 353)^d^	73 (20.7)	5 (16.7)	4 (11.1)	2 (9.1)	4 (26.7)	6 (42.9)	5 (38.5)	2 (18.2)	1 (9.1)	0	4 (50)	0	40 (23.3)	0.024
Ceftazidime (*n* = 409)^d^	167 (40.8)	15 (34.9)	26 (65)	5 (21.7)	9 (50)	10 (58.8)	8 (50)	3 (23.1)	3 (27.3)	3 (25)	5 (45.5)	2 (20)	78 (40)	0.018
Cefuroxime (*n* = 358)^d^	242 (67.6)	17 (53.1)	32 (84.2)	9 (39.1)	12 (70.6)	16 (100)	11 (91.7)	7 (100)	6 (54.5)	6 (50)	6 (75)	10 (100)	110 (64)	<0.001
Ertapenem (*n* = 119)^d^	3 (2.5)	0	0	0	0	0	0	0	0	0	0	ND	3 (5.0)	1.000
Imipenem (*n* = 299)^d^	7 (2.3)	2 (5.4)	0	0	0	0	0	0	1 (14.3)	0	0	0	4 (2.9)	0.716
Meropenem (*n* = 309)^d^	7 (2.3)	2 (5.4)	0	0	0	0	0	0	0	0	0	1 (10)	4 (2.8)	0.781
Trimethoprim/sulfamethoxazole (*n* = 403)^d^	285 (70.7)	26 (60.5)	27 (69.2)	19 (82.6)	14 (87.5)	15 (100)	12 (75.0)	10 (76.9)	9 (75)	8 (66.7)	7 (63.6)	7 (70)	131 (67.9)	0.179
Nitrofurantoin (*n* = 139)^d^	14 (10.1)	3 (21.4)	1 (7.1)	0	ND	0	0	0	0	0	2 (100)	0	8 (13.1)	0.092
Ciprofloxacin (*n* = 374)^d^	268 (71.7)	40 (100)	39 (97.5)	7 (41.2)	12 (75)	17 (100)	6 (40)	3 (27.3)	9 (81.8)	7 (63.6)	10 (100)	0	118 (67)	<0.001
Levofloxacin (*n* = 410)^d^	275 (67.1)	43 (100)	39 (97.5)	7 (30.4)	13 (72.2)	16 (94.1)	5 (31.3)	3 (23.1)	10 (83.3)	7 (58.3)	11 (100)	1 (10)	120 (61.5)	<0.001
Minocycline (*n* = 212)^d^	76 (35.8)	7 (22.6)	4 (19)	4 (44.4)	0	8 (80)	4 (36.4)	1 (14.3)	0	2 (22.2)	1 (16.7)	9 (90)	36 (40)	<0.001
Tetracycline (*n* = 200)^d^	161 (80.5)	17 (73.9)	19 (82.6)	7 (77.8)	4 (80)	11 (100)	10 (90.9)	0	5 (83.3)	8 (72.7)	2 (50)	10 (100)	68 (81.9)	0.013
ESBL phenotype (*n* = 313)^d^	184 (58.8)	11 (44)	23 (71.9)	7 (31.8)	10 (83.3)	13 (86.7)	7 (63.6)	7 (77.8)	6 (66.7)	6 (60)	3 (42.9)	10 (100)	81 (53.6)	0.001
MDR	272 (66.2)	27 (62.8)	36 (90)	12 (52.2)	13 (72.2)	17 (100)	12 (75)	4 (30.8)	11 (91.7)	8 (66.7)	7 (63.6)	8 (80)	117 (59.7)	<0.001
Resistance score^e^, median (IQR)	8 (3.5-13)	7 (3–13)	10.3 (7.1–14.9)	6 (2.5–8.5)	8 (2.8–10.6)	13 (8.5–16.3)	12.3 (5.1–14.4)	7.5 (2.8–8.8)	7.3 (5.3–13.4)	7.8 (5.6–12.8)	6.5 (3.5–14)	10.5 (8.9–12)	7.8 (2.6–12.9)	0.001

*^*a*^For *fimH*-null isolates (isolates without *fimH*), MLST was performed according to the Achtman scheme using seven housekeeping genes (Wirth et al., 2006). ^*b*^Other isolates (196) include CH11-27 (9); CH4-24 and CH24-10 (8 each); CH4-27 and CH65-32 (7 each); CH4-58 (6); CH4-32 (5); CH4-35, CH4-54, CH4-61, CH11-34, CH88-145, ST167, and ST773 (4 each); CH4-29, CH7-34, CH24-30, CH29-38, CH33-44, CH40-20, and CH41-86 (3 each); CH6-35, CH7-41, CH11-23, CH11-24, CH11-30, CH13-41, CH19-86, CH19-87, CH23-31, CH37-29, CH38-5, CH40-22, CH106-54, CH165-27, and ST617 (2 each); CH3-299, CH4-31, CH4-34, CH4-39, CH4-121, CH4-142, CH6-31, CH6-32, CH7-27, CH7-54, CH11-25, CH11-29, CH11-69, CH11-398, CH11-419, CH11-475, CH11-946, CH13-106, CH13-130, CH13-143, CH13-429, CH13-1488, CH14-4, CH14-27, CH23-25, CH24-54, CH26-49, CH27-23, CH27-41, CH29-1127, CH35-47, CH35-54, CH36-54, CH37-263, CH38-15, CH40-89, CH40-99, CH45-97, CH45-453, CH52-5, CH52-14, CH67-222, CH88-54, CH88-58, CH88-1010, CH132-27, CH184-108, CH834-47, ST10, ST31, ST227, ST354, ST450, ST501, ST1284, and ST1290 (1 each), and 11 new genotypes. ^*c*^Results were interpreted according to CLSI (2019) guidelines, except for cefoperazone/sulbactam interpreted using cefoperazone CLSI breakpoints and tetracycline were interpreted by U.S. Food and Drug Administration breakpoints. For each antimicrobial, resistance is defined by the sum of resistant or intermediary isolates. ND, not detected; MDR, Multidrug-resistant; ESBL, extended-spectrum beta-lactamase; IQR, interquartile range. ^*d*^Not all isolates were tested against all agents, accounting for the smaller sample size in some rows. ^*e*^Resistance score was defined as the sum of inactive *in vitro* antimicrobial agents for each isolate. A score of 1 was attributed for a resistant, 0.5 for an intermediary, and 0 for a sensitive isolate; a higher score thus indicated a more resistant isolate.*

Acquired ARGs that conferred antibiotic resistance were further analyzed based on the genome sequences of 74 representative strains. *Bla*_CMY__–__2_, *bla*_CTX–M_ (*bla*_CTX–M–__3_, *bla*_CTX–M__–__14_, *bla*_CTX–M__–__15_, etc.), *bla*_OXA–__1_, *bla*_TEM__–__1__*B*_ were detected in 3 (4.1%), 48 (64.9%), 12 (16.2%), and 40 (54.1%) isolates, respectively (Supplementary Table S2). *Bla*_TEM__–__1__*B*_ and *bla*_CMY__–__2_ could be responsible for multi-antibiotics (ampicillin, piperacillin, and cefazolin) and cefoxitin resistance respectively (Bauernfeind et al., 1996; Sun et al., 2013). *Bla*_OXA–__1_ could confer low-level resistance to cefepime (Antunes and Fisher, 2014). *Bla*_CTX–M_ genes could be responsible for resistance to ampicillin, piperacillin, aztreonam, cefatriaxone, cefazolin, cefepime, cefotaxime, ceftazidime, and cefuroxime (Bonnet, 2004), which played a major role in the β-lactam resistance of ExPEC isolates. In addition, *aac(6′)-Ib-cr* was detected in 13 (17.6%) isolates (Supplementary Table S2) and reported to only cause low-level ciprofloxacin resistance (Robicsek et al., 2006). *TetA* and *tetB* were detected in 31 (41.9%) and 17 (23%) of 74 isolates respectively (Supplementary Table S2) and could be responsible for tetracycline resistance (Shales et al., 1980). *Sul* and *dfrA* were detected in 38 (51.4%) and 34 (45.9%) of 74 isolates respectively (Supplementary Table S2) and could confer resistance to trimethoprim/sulfamethoxazole (Hu et al., 2011).

### Lethal *G. mellonella* Infection

**FIGURE 1:**
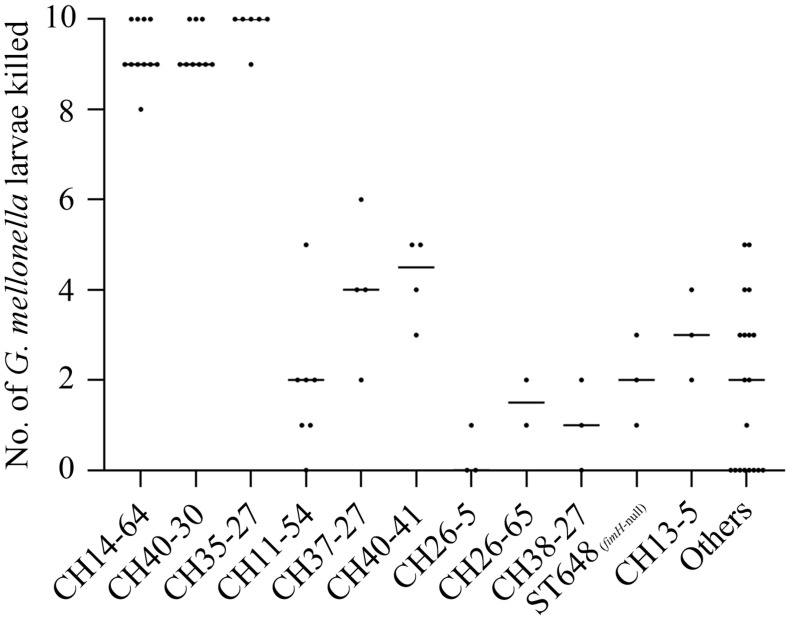
The number of larvae killed in a *G. mellonella* infection model according to CH or ST types among the 74 ExPEC isolates. Lethality in larvae (10 larvae/*E. coli* strain) was observed for 4 days after infection with test strains. Each dot corresponds to one test strain, its corresponding abscissa represents the average number of three independent experiments for the strain, and its corresponding ordinate represents the CH or ST types of the strain. Non-infected larvae and PBS challenged larvae showed no mortality. Low larvae mortality (≤20%) was recorded following challenge with *E. coli* DH5α. The Mann–Whitney *U* test showed the number of larvae killed in CH14-64, CH40-30, and CH35-27 groups were not statistically different, but higher than that in any of the remaining groups (*P* < 0.05).

Larvae inoculated with CH14-64-ST1193, CH40-30-ST131, and CH35-27-ST69 isolates showed significantly higher mortality rates (*P* < 0.05) than those infected with CH11-54, CH37-27-ST405, CH40-41-ST131, CH26-5-ST38, CH26-65, CH38-27-ST95, ST648 (*fimH*-null), CH13-5-ST12 and other isolates in the infection model, killing ≥80% (8/10) of the larvae within 96 h (Figure 1). The above-mentioned results indicated that B2-CH14-64 (CH14-64 isolates belonging to B2), B2-CH40-30, and D-CH35-27 isolates were highly virulent in these molecular types. However, B2-CH40-41, B2-CH13-5, and D-CH37-27 isolates exhibited medium virulence killing 20–60% (2–6/10) of the larvae. B2-CH38-27, D-CH26-5 and D-CH26-65 isolates only killed ≤20% (2/10) of the larvae, displaying low virulence. A-CH11-54 (with one exception) and F-ST648 (*fimH*-null) isolates were also of low virulence and killed ≤30% (3/10) of the larvae. In addition, the Mann–Whitney U test showed the number of larvae killed by ExPEC from different sources (urine, sputum, blood, throat swab, and others) (Supplementary Table S1) were not statistically different (data not shown), which might be because most of the 74 tested strains were from urine (66.2%).

The difference between each of all of the detected VFs (Supplementary Table S3) and corresponding numbers of larvae killed was analyzed using Mann–Whitney *U*-test to further explore key VFs in the infection model (Table 2). ExPEC isolates carrying VFs involved in adherence (*papB*, *papI*, *papX*, and *fimA*), autotransporter (*sat*), invasion (*aslA* and *kpsD*), iron uptake (as in Table 2, except for *entD*), or toxin (*senB*) killed more larvae than corresponding VF negative strains (*P* < 0.05). The above-mentioned results indicated that these VFs played an important role in the model. Unusually, ExPEC isolates carrying VFs involved in adherence (*afaA*, *afaB-I*, *afaC-I*, *draP*, and *daaF*), iron uptake (only *entD*), or secretion system (as in Table 2) killed less larvae than corresponding VF negative strains (*P* < 0.05). The results of VFs involved in adherence (*afaA*, *afaB-I*, *afaC-I*, *draP*, and *daaF*) might not be reliable because they only had 2–3 (2.7–4.1%) positive strains (Table 2). Each of the 74 ExPEC isolates was found to carry 39-121 VFs (Supplementary Table S3) and infection was not linked to a single VF but rather to multiple traits (Johnson et al., 2006; Ciesielczuk et al., 2015; Johnson et al., 2019a). VFs involved in iron uptake (only *entD*) or secretion system (as in Table 2) might play a very weak role in the model.

**TABLE 2:** Associations between the number of larvae killed (experimental virulence) and the presence of a virulence factor (VF) among the 74 ExPEC strains.

**VFs^a^**		**VF^+^**		**VF^–^**		***P***
		**No. of VF^+^ strains**	**No. of larvae killed, median (IQR)^b^**	**No. of VF^–^ strains**	**No. of larvae killed, median (IQR)^b^**	
Adherence						
Afimbrial adhesin AFA-I	*afaA*	2	(0–0)^c^	72	4 (2–9)	0.037
	*afaB-I*	3	0 (0–0)^d^	71	4 (2–9)	0.038
	*afaC-I*	3	0 (0–0)^d^	71	4 (2–9)	0.038
Dr. adhesins	*draP*	3	0 (0–0)^d^	71	4 (2–9)	0.038
F1845 fimbrial adhesin	*daaF*	3	0 (0–0)^d^	71	4 (2–9)	0.038
P fimbriae	*papB*	48	7 (2.3–9)	26	2 (0–3)	<0.001
	*papI*	46	8.5 (3–9)	28	2 (0–3)	<0.001
	*papX*	55	5 (2–9)	19	2 (1–3)	0.015
Type 1 fimbriae	*fimA*	52	7 (3–9)	22	1.5 (0–2.3)	<0.001
Autotransporter						
Sat	*sat*	34	9 (4–9.3)	40	2 (1–4)	<0.001
Invasion						
AslA	*aslA*	67	4 (2–9)	7	0 (0–3)	0.015
K1 capsule	*kpsD*	51	5 (2–9)	23	2 (1–4)	0.019
Iron uptake						
Aerobactin	*iucA*	48	5 (2–9)	26	2 (1–4.3)	0.024
	*iucB*	48	5 (2–9)	26	2 (1–4.3)	0.024
	*iucC*	48	5 (2–9)	26	2 (1–4.3)	0.024
	*iutA*	50	5.5 (2–9)	24	2 (1–3.8)	0.002
Chu	*chuA*	50	8.5 (2.8–9.3)	24	1.5 (0–3)	< 0.001
	*chuS*	55	5 (2–9)	19	2 (0–3)	<0.001
	*chuT*	44	5 (2.3–9)	30	2 (0–4.3)	0.004
	*chuU*	56	5 (2–9)	18	1 (0–2.3)	<0.001
	*chuV*	57	5 (2–9)	17	1 (0–2.5)	<0.001
	*chuW*	57	5 (2–9)	17	1 (0–2.5)	< 0.001
	*chuX*	45	5 (2–9)	29	2 (0–5.5)	0.019
	*chuY*	56	5 (2–9)	18	1 (0–2.3)	<0.001
Shu	*shuU*	37	6 (2–9.5)	37	2 (0–5)	0.003
Enterobactin	*entD*	49	2 (0.5–4)	25	9 (8.5–10)	<0.001
Yersiniabactin	*fyuA*	59	5 (2–9)	15	2 (0–3)	0.008
	*irp1*	59	5 (2–9)	15	2 (0–3)	0.008
	*irp2*	58	5 (2–9)	16	1.5 (0–3)	0.008
	*ybtA*	59	5 (2–9)	15	2 (0–3)	0.008
	*ybtE*	59	5 (2–9)	15	2 (0–3)	0.008
	*ybtP*	59	5 (2–9)	15	2 (0–3)	0.008
	*ybtQ*	59	5 (2–9)	15	2 (0–3)	0.008
	*ybtS*	59	5 (2–9)	15	2 (0–3)	0.008
	*ybtT*	59	5 (2–9)	15	2 (0–3)	0.008
	*ybtU*	59	5 (2–9)	15	2 (0–3)	0.008
	*ybtX*	59	5 (2–9)	15	2 (0–3)	0.008
Secretion system						
T3SS	*espL1*	41	2 (0–4)	33	9 (4–9)	<0.001
	*espL4*	28	2 (1–4.8)	46	5 (2–9)	0.015
	*espX1*	38	2 (0–4)	36	9 (3–9)	0.001
	*espX4*	41	2 (0–4)	33	9 (4–9)	<0.001
	*espX5*	36	2 (0–4)	38	8.5 (3–9)	0.001
	*espY1*	32	2 (1–4.8)	42	5 (2.8–9)	0.014
	*espY2*	16	2 (1–3.8)	58	5 (2–9)	0.007
T2SS	*gspD*	55	3 (1–9)	19	9 (2–10)	0.044
	*gspE*	55	3 (1–9)	19	9 (2–10)	0.044
	*gspF*	55	3 (1–9)	19	9 (2–10)	0.044
	*gspG*	55	3 (1–9)	19	9 (2–10)	0.044
	*gspH*	55	3 (1–9)	19	9 (2–10)	0.044
	*gspJ*	55	3 (1–9)	19	9 (2–10)	0.044
	*gspK*	56	3 (1–8.8)	18	9 (2.8–10)	0.014
	*gspL*	64	3 (1–9)	10	9.5 (4.3–10)	0.021
	*gspM*	63	3 (1–9)	11	9 (5–10)	0.012
Toxin						
ShET2	*senB*	28	9 (4–10)	46	2 (1–5)	0.001

*^*a*^A VF was listed in the table when Mann–Whitney *U*-test showed that number of larvae killed between VF-positive strains and VF-negative strains were statistically different (*P* < 0.05). ^*b*^IQR, interquartile range. ^*c*^(min–max). ^*d*^Median (min–max).*

### Gene (ARGs and VFs) Capacity and Subtype Specificity

**FIGURE 2:**
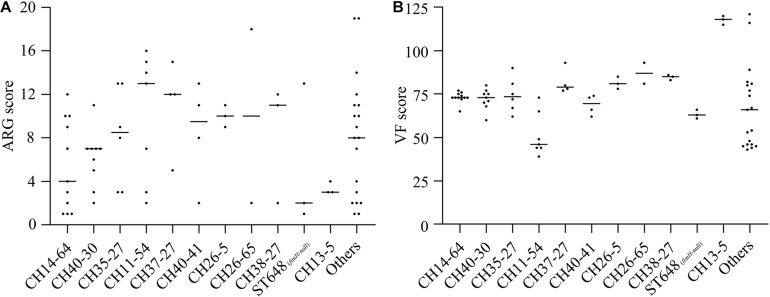
ARG **(A)** and VF **(B)** scores according to CH or ST types among the 74 ExPEC isolates. ARG and VF scores were defined as the number of unique ARG and VF detected for each isolate, respectively. ARG scores in CH37-27 isolates were significantly higher than in CH14-64 or CH13-5 isolates while ARG scores in CH26-5 isolates were significantly higher than in CH40-30 or CH13-5 isolates (*P* < 0.05). VF scores in CH13-5 isolates were significantly higher than in any of these 12 groups (except CH26-65) while VF scores in CH11-54 isolates were significantly lower than in any of these 12 groups (except ST648 and Others) (*P* < 0.05).

We further analyzed ARG and VF scores of different types of strains to explore whether ARGs and VFs were correlated with intensities of resistance and virulence. Among these molecular types, CH37-27-ST405 and CH26-5-ST38 isolates carried more ARGs while CH13-5-ST12 isolates carried fewer ARGs; CH13-5-ST12 isolates carried the most VFs, CH37-27-ST405, CH26-5-ST38, and CH38-27-ST95 isolates carried more VFs, and CH11-54 isolates carried the least VFs (Figure 2, Supplementary Table S4). However, CH40-30-ST131, CH37-27-ST405, CH40-41-ST131, and CH13-5-ST12 isolates were extensively drug-resistant (Table 1) while CH14-64-ST1193, CH40-30-ST131, and CH35-27-ST69 isolates were highly virulent (Figure 1). These types of strains with high ARGs or VFs capacities hardly showed extensive drug-resistance or high virulence. This was primarily because the tested strains harbored β-lactam resistance and the *G. mellonella* killing assay did not fully correlate with all detected ARGs and VFs. Differential analysis of ARGs and VFs according to molecular types among the 74 ExPEC isolates was also done to explain their phenotype (Supplementary Table S4). For β-lactam and fluoroquinolone resistance, *bla*_CTX–M–__15_ and *aac(6′)-Ib-cr* were more prevalent in CH40-30 isolates than in non-CH40-30 isolates (*P* < 0.05). For tetracycline resistance, *tetB* in CH35-27-ST69 and CH13-5-ST12 isolates (there was no significant difference between them) were more prevalent than in any of the remaining groups (*P* < 0.05). Each of the 74 ExPEC isolates was found to harbor 1–19 acquired ARGs and 39–121 VFs, which suggests that ExPEC may have greater genome plasticity (Supplementary Tables S2, S3). VFs involved in adherence (*papB*, *papI*, *papX*, and *fimA*), autotransporter (*sat*), invasion (*kpsD*), iron uptake (as in Table 2, except for *entD*), and toxin (*senB*) were responsible for pathogenicity *in vivo*. Of these VFs, *papB*, *papI*, *fimA*, *sat*, *kpsD*, *senB*, and aerobactin genes in CH14-64-ST1193 isolates were significantly higher than in non-CH14-64-ST1193 isolates; *papB*, *papI*, *sat*, *iucA*, *iucB*, *iucC*, *chuT*, *chuX*, and *shuU* in CH40-30-ST131 isolates were significantly higher than in non-CH40-30-ST131 isolates; but only *chuX* in CH35-27-ST69 isolates was significantly lower than in non-CH35-27-ST69 isolates. Most of the VFs responsible for pathogenicity, including *papB*, *papI*, *papX*, *fimA*, *kpsD*, *chu*, and yersiniabactin genes, in CH11-54 isolates were significantly lower than in non-CH11-54 isolates. 100% of ST648 (*fimH*-null) isolates did not contain VFs involved in P fimbriae (as in Supplementary Table S4, except for *papX*), type 1 fimbriae (Supplementary Table S4), and iron uptake [aerobactin genes (*iucA*, *iucB*, *iucC*, and *iutA*)]. These differential VFs between target and non-target types were partly responsible for the virulence phenotypes of the isolates.

## Discussion

CH typing based on the sequencing of two loci (*fumC*/*fimH*) is faster and cheaper than the standard seven-locus MLST, and specific CH types correspond to specific STs with 95% accuracy (Roer et al., 2018). Therefore, we used CH typing to evaluate the 411 ExPEC isolates while MLST supplemented with CH typing was only performed for *fimH*-null isolates. The major clonal groups (CH14-64-ST1193, CH40-30-ST131, CH40-41-ST131, CH35-27-ST69, CH37-27-ST405, CH26-5-ST38, CH38-27-ST95, ST648, and CH13-5-ST12) in this study were also globally dominant ExPEC lineages (Manges et al., 2019).

Among these types, CH40-30-ST131, CH37-27-ST405, CH40-41-ST131, and CH13-5-ST12 isolates had high resistance rates to all tested antimicrobials containing 17 (65.4%) β-lactam antibiotics (Table 1). This is consistent with ST131 and ST405 isolates associated with ESBLs and MDR (Pitout, 2012; Manges et al., 2019) but is not compatible with ST12 isolates exhibiting lower MDR levels (Manges et al., 2019). This is possibly related to limitations of our CH13-5-ST12 strains mostly isolated from sputum samples of infants with bronchopneumonia (2011.12-2012.04) (Supplementary Table S5). In addition, CH14-64-ST1193 and CH40-30-ST131 isolates with a high frequency of fluoroquinolone resistance are consistent with previous studies (Nicolas-Chanoine et al., 2014; Johnson et al., 2019b). CH37-27-ST405 and ST648 isolates also had high resistance rates to fluoroquinolones. There were higher resistance rates among our isolates compared with data from the China antimicrobial resistance surveillance system^4^ (CARSS) from 2014 to 2018, which indicated resistance for third-generation cephalosporins [cefatriaxone or cefotaxime, 59.7% (2014), 59% (2015), 56.6% (2016), 54.2% (2017), and 53% (2018)] and fluoroquinolones [ciprofloxacin or levofloxacin, 54.3% (2014), 53.5% (2015), 52.9% (2016), 51% (2017), and 50.8% (2018)]. This was because some of our isolates were collected from 2011 to 2012 [cefatriaxone or cefotaxime, 68.1% (2011–2012) and 47.7% (2018); ciprofloxacin or levofloxacin, 70.6% (2011–2012) and 62.9% (2018)]. In recent years, the resistance rates of ExPEC to third-generation cephalosporins and fluoroquinolones in China have showed a slight downward trend, but they are still relatively high (>50%). Resistance rates in China are higher than in most countries in the world [third-generation cephalosporins and fluoroquinolones: America (14.6% and 33.3%), Japan (16.6% and 34.3%), Germany (8% and 23.7%), France (8.2% and 17.9%), United Kingdom (9.6% and 17.5%), etc.] (Collignon and McEwen, 2019). The differences in antibiotic resistance may be caused by antibiotic usage, local temperatures, population densities, and other environmental factors (Fletcher, 2015; MacFadden et al., 2018).

Among these types, CH14-64-ST1193, CH40-30-ST131, and CH35-27-ST69 isolates showed high virulence in the infection model. ST69 isolates were reported to show higher virulence than ST95 and ST131 isolates (Alghoribi et al., 2014) while our research specifically showed that ST131-H30 (H30 subclone of sequence type 131) and ST69 isolates (both having equal virulence) had higher virulence than ST131-H41 and ST95 isolates. Compared with PCR surveillance of 29-55 VFs in previous similar studies (Johnson et al., 2006; Alghoribi et al., 2014; Merino et al., 2020), we detected the known VFs in VFDB to investigate VFs responsible for pathogenicity *in vivo*. Consistent with the literature (Johnson et al., 2006), *pap* (P fimbriae elements) and *fyuA* were closely associated with the virulence of ExPEC. Other key VFs (Table 2) associated with the virulence of ExPEC in this study were different from reports in previous studies (Johnson et al., 2006; Ciesielczuk et al., 2015; Johnson et al., 2019a). The difference is more likely to come from the differences of infection models and detected VFs between this study and these previous investigations. Apart from *sat* and *senB* which were frequently detected in ExPEC, a large number of VFs involved in iron uptake (as in Table 2, except for *entD*) were identified as playing a role in the experimental virulence of ExPEC. These VFs might play a more important role in pathogenicity *in vivo*. Besides, a large number of VFs involved in secretion system were detected and supposed to be negatively associated with the virulence of ExPEC. Some (*espL*, *espR*, *espX*, and *espY*) of these VFs encoded type III secretion system (T3SS) effectors in enterohemorrhagic *E. coli* (EHEC) O157:H7 (Tobe et al., 2006) and others (*gspC*, *gspD*, *gspE*, *gspF*, *gspG*, *gspH*, *gspI*, *gspJ*, *gspK*, *gspL*, and *gspM*) probably encoded secretory pathway proteins of type II secretion system (T2SS) in *Shigella dysenteriae* Sd197 (Yang et al., 2005). The products of these VFs were effectors and secretory pathway proteins, these components could not form a complete T3SS or T2SS, and they could not perform the function of T3SS or T2SS (Green and Mecsas, 2016). This might be a main reason that these VFs involved in T3SS or T2SS were negatively correlated with the virulence of ExPEC. Moreover, these VFs involved in T3SS or T2SS did not perfectly matched (100% identity and 100% coverage) with corresponding reference genes in VFDB, their nucleotide identities ranged from 90.1 to 99% (data not shown), and the small variations might disrupt the function of these VFs. This might be another reason for these VFs negatively correlated with the virulence of ExPEC. Further studies are needed to determine the functions of these above-mentioned VFs.

We also analyzed ARG and VF scores, differential ARGs and VFs according to molecular types among the 74 ExPEC isolates to explain their phenotype (Supplementary Table S4). Compared with previous study on ST131 strains producing *bla*_CTX–M–__15_ and *aac(6′)-Ib-cr* (Alsharapy et al., 2018), this study clearly indicated that a high percentage of *bla*_CTX–M–__15_ and *aac(6′)-Ib-cr* was detected in CH40-30-ST131 isolates but not in isolates belonging to other molecular types. The prevalence of *tetB* in CH35-27-ST69 and CH13-5-ST12 isolates was also reported in this study. Currently, available studies with which to compare differential VFs according to molecular types are scarce. In this study, VFs differed according to molecular types, such as CH14-64-ST1193 isolates with a high prevalence of *papB*, *papI*, *papX*, *sat*, *senB*, aerobactin and yersiniabactin genes; CH40-30-ST131 isolates with a high prevalence of *sat*, aerobactin and yersiniabactin genes; CH35-27-ST69 isolates with a high percentage of yersiniabactin genes; CH11-54 isolates with a low percentage of VFs involved in K1 capsule (*kps*), and iron uptake (*chu* and yersiniabactin genes). These VFs might be responsible for the experimental virulence of different types of strains. This suggests that the key VFs are probably different in the same pathogenic process induced by different types of strains. ARG and VF scores according to molecular types were analyzed in this study. Perhaps more significantly, CH37-27-ST405 and CH26-5-ST38 isolates carried more ARGs and VFs among these molecular types, which meant that they had a high resistance and virulence potential and might become more widespread in the future.

In summary, among 411 ExPEC isolates, CH40-41-ST131 and CH13-5-ST12 isolates exhibited a high prevalence of resistance to third-generation cephalosporins, CH14-64-ST1193 and ST648 (*fimH*-null) isolates had high resistance rates to fluoroquinolones, CH40-30-ST131 and CH37-27-ST405 isolates simultaneously exhibited high resistance to third-generation cephalosporins and fluoroquinolones. Meanwhile, CH14-64-ST1193, CH40-30-ST131, and CH35-27-ST69 isolates showed high virulence in the infection model. With regards to genotypes, *bla*_CTX–M_ played a major role in the β-lactam resistance of ExPEC isolates while VFs involved in adherence (*papB*, *papI*, *papX*, *fimA*), autotransporter (*sat*), invasion (*aslA*, *kpsD*), iron uptake (as in Table 2, except for *entD*), or toxin (*senB*) might be responsible for pathogenicity *in vivo*, especially a large number of VFs involved in iron uptake. Specific ARGs or VFs were prevalent in specific types of strains, including *papB*, *papI*, *fimA*, *sat*, *kpsD*, *senB*, and aerobactin genes in CH14-64-ST1193 isolates; *bla*_CTX–M–__15_, *aac(6′)-Ib-cr*, *papB*, *papI*, *sat*, *iucA*, *iucB*, *iucC*, *chuT*, *chuX*, and *shuU* in CH40-30-ST131 isolates; *tetB* in CH35-27-ST69 and CH13-5-ST12 isolates. Type distribution also differed by VF score. Perhaps more significantly, CH37-27-ST405 and CH26-5-ST38 isolates carried more ARGs and VFs among these molecular types, which indicated that they had a high resistance and virulence potential. This study also has some limitations including the reliance on a *G. mellonella* infection model, analysis only of elected bacterial traits (and for presence or absence only), and small number in some subgroups. Further studies are required to strengthen the analysis of type-specific ARGs and VFs and to fully understand their function in resistance and virulence.

## Data Availability Statement

The datasets presented in this study can be found in online repositories. The names of the repository/repositories and accession number(s) can be found in the article/Supplementary Material.

## Author Contributions

YL, DM, YD, and HR conceived, coordinated the study, and provided the financial support. SL, YC, YD, SZ, HG, and LZ collected the strains and did antimicrobial susceptibility testing. HG, LZ, and YD carried out CH (or ST) typing, whole-genome sequencing, and the *G. mellonella* killing assay. YD, SL, HG, and ZW analyzed the data. YD drafted the manuscript. YL, DM, and HR revised the manuscript. All authors read and approved the final manuscript.

## Conflict of Interest

The authors declare that the research was conducted in the absence of any commercial or financial relationships that could be construed as a potential conflict of interest.

## Footnotes

1 https://cge.cbs.dtu.dk/services/chtyper/

2 https://cge.cbs.dtu.dk/services/MLST/

3 http://clermontyping.iame-research.center/

4 http://www.carss.cn/
