# Supplementary material for: Antibiotic Resistance and Virulence of Extraintestinal Pathogenic Escherichia coli (ExPEC) Vary According to Molecular Types
Source: Front Microbiol. 2020 Nov 25;11:598305. doi: 10.3389/fmicb.2020.598305 (PMC7732638; doi:10.3389/fmicb.2020.598305)
Supplement: Supplementary file 2 [file Data_Sheet_2.docx]

**TABLE S5** Sources of the 411 ExPEC isolates according to CH or ST types.

| Sources | No. (%) of isolates | | | | | | | | | | | | | *P* |
| --- | --- | --- | --- | --- | --- | --- | --- | --- | --- | --- | --- | --- | --- | --- |
|  | All  (n = 411) | CH14-64  (n = 43) | CH40-30  (n = 40) | CH35-27  (n = 23) | CH11-54  (n = 18) | CH37-27  (n = 17) | CH40-41  (n = 16) | CH26-5  (n = 13) | CH26-65  (n = 12) | CH38-27  (n = 12) | ST648^a^  (n = 11) | CH13-5  (n = 10) | Others^b^  (n = 196) |  |
| Urine | 261 (63.5) | 34^c^ (79.1) | 18^c^ (45) | 18 (78.3) | 12 (66.7) | 13 (76.5) | 9 (56.3) | 7 (53.8) | 11 (91.7) | 5 (41.7) | 7 (63.6) | 0^c^ | 127 (64.8) | < 0.001 |
| Sputum | 64 (15.6) | 1^c^ (2.3) | 8 (20) | 2 (8.7) | 1 (5.6) | 3 (17.6) | 2 (12.5) | 0 | 0 | 4 (33.3) | 1 (9.1) | 9^c^ (90) | 33 (16.8) |  |
| Blood | 41 (10) | 5 (11.6) | 8^c^ (20) | 1 (4.3) | 1 (5.6) | 1 (5.9) | 4 (25) | 1 (7.7) | 1 (8.3) | 2 (16.7) | 2 (18.2) | 0 | 15 (7.7) |  |
| Throat swab | 15 (3.6) | 1 (2.3) | 4^c^ (10) | 0 | 1 (5.6) | 0 | 0 | 1 (7.7) | 0 | 1 (8.3) | 0 | 0 | 7 (3.6) |  |
| Others | 30 (7.3) | 2 (4.7) | 2 (5) | 2 (8.7) | 3 (16.7) | 0 | 1 (6.3) | 4^c^ (30.8) | 0 | 0 | 1 (9.1) | 1 (10) | 14 (7.1) |  |

^a^For *fimH*-null isolates (isolates without *fimH*), MLST was performed according to the Achtman scheme using seven housekeeping genes (Wirth et al., 2006).

^b^Other isolates (196) include CH11-27 (9); CH4-24 and CH24-10 (8 each); CH4-27 and CH65-32 (7 each); CH4-58 (6); CH4-32 (5); CH4-35, CH4-54, CH4-61, CH11-34, CH88-145, ST167, and ST773 (4 each); CH4-29, CH7-34, CH24-30, CH29-38, CH33-44, CH40-20, and CH41-86 (3 each); CH6-35, CH7-41, CH11-23, CH11-24, CH11-30, CH13-41, CH19-86, CH19-87, CH23-31, CH37-29, CH38-5, CH40-22, CH106-54, CH165-27, and ST617 (2 each); CH3-299, CH4-31, CH4-34, CH4-39, CH4-121, CH4-142, CH6-31, CH6-32, CH7-27, CH7-54, CH11-25, CH11-29, CH11-69, CH11-398, CH11-419, CH11-475, CH11-946, CH13-106, CH13-130, CH13-143, CH13-429, CH13-1488, CH14-4, CH14-27, CH23-25, CH24-54, CH26-49, CH27-23, CH27-41, CH29-1127, CH35-47, CH35-54, CH36-54, CH37-263, CH38-15, CH40-89, CH40-99, CH45-97, CH45-453, CH52-5, CH52-14, CH67-222, CH88-54, CH88-58, CH88-1010, CH132-27, CH184-108, CH834-47, ST10, ST31, ST227, ST354, ST450, ST501, ST1284, and ST1290 (1 each), and 11 new genotypes.

^c^*P* value was < 0.05 in comparison with all of the other isolates.
